# Joint Placement and Device Association of UAV Base Stations in IoT Networks

**DOI:** 10.3390/s19092157

**Published:** 2019-05-09

**Authors:** Ashfaq Ahmed, Muhammad Awais, Tallha Akram, Selman Kulac, Musaed Alhussein, Khursheed Aurangzeb

**Affiliations:** 1 Department of Electrical & Computer Engineering, COMSATS University Islamabad, Wah Campus, Wah Cantt 47040, Pakistan; ashfaqahmed@ciitwah.edu.pk (A.A.); tallha@ciitwah.edu.pk (T.A.); 2 Department of Electrical-Electronics Engineering, Faculty of Engineering, Duzce University, Konuralp, Duzce 81620, Turkey; selmankulac@duzce.edu.tr; 3 Computer Engineering Department, College of Computer and Information Sciences, King Saud University, Riyadh 11543, Saudi Arabia; musaed@ksu.edu.sa

**Keywords:** resource management, Unmanned aerial vehicles (UAVs), Aerial base stations, Internet of Things (IoTs)

## Abstract

Drone base stations (DBSs) have received significant research interest in recent years. They provide a flexible and cost-effective solution to improve the coverage, connectivity, quality of service (QoS), and energy efficiency of large-area Internet of Things (IoT) networks. However, as DBSs are costly and power-limited devices, they require an efficient scheme for their deployment in practical networks. This work proposes a realistic mathematical model for the joint optimization problem of DBS placement and IoT users’ assignment in a massive IoT network scenario. The optimization goal is to maximize the connectivity of IoT users by utilizing the minimum number of DBS, while satisfying practical network constraints. Such an optimization problem is NP-hard, and the optimal solution has a complexity exponential to the number of DBSs and IoT users in the network. Furthermore, this work also proposes a linearization scheme and a low-complexity heuristic to solve the problem in polynomial time. The simulations are performed for a number of network scenarios, and demonstrate that the proposed heuristic is numerically accurate and performs close to the optimal solution.

## 1. Introduction

The use of drone base stations (DBSs) has been realized as a promising addition to the conventional wireless networks. DBSs are adopted to achieve multiple objectives. On one hand, they help to increase the coverage of existing terrestrial networks, such as broadband and cellular networks [1]. On the other hand, they serve as assisting relays to improve the connectivity of ground wireless devices [2]. In contrast to the terrestrial BSs which are fixed, DBSs have the ability to fly and deliver network services to any hard-to-reach areas. In addition to the horizontal manoeuvring, they have the ability to adapt their altitude. This enables them to establish LOS links to ground users [3]. Moreover, they improve the capacity of networks by serving as mobile hotspots. Along with the open-air communication, small drones have also proven to be an effective solution for indoor communications [4].

Internet of Things (IoT) is one exciting application of DBSs. IoT refers to a set of small, uniquely identifiable devices connected to the Internet. Practical IoT networks are composed of a massive number of heterogenous devices, which include smart phones, sensors, electronic gadgets and wearables, network connectivity modules implanted on the vehicles, animals, household electrical appliances, medical equipment, and many more. These devices perform tasks in a diverse range of applications, such as E-health [5], personal healthcare devices [6], radio sensor networks [7], the intelligent transport system (ITS) [8], smart cities [9], and industrial IoT [10]. The massive nature of IoT poses some major challenges, including spectrum scarcity, reliability, energy efficiency, high-speed uplinks, and ultra-low latency. In particular, the IoT devices are highly power constrained and lack the ability to communicate at longer distances. This becomes a critical issue when these devices are deployed in areas with poor or no coverage provided by existing terrestrial wireless networks.

In such an IoT scenario, the use of DBSs can be a promising solution. The DBSs can serve the role of moving data aggregators—in other words, they can fly close to IoT devices, collect their data, and transmit it to other devices which are out-of-range of the transmitting IoT devices. In addition, due to their aerial nature, DBSs can be deployed at high altitudes. This helps to mitigate the shadowing effects and increases the probability of LOS communication between the DBSs and ground IoT devices. Consequently, the battery-limited IoT devices will be able to communicate with much lower transmit power. In addition, DBSs also have the ability to change their location based on the activation pattern of IoT devices. This helps them to support an anticipated number of devices in massive IoT deployment.

### 1.1. Literature Review

Despite the elegant features, DBSs themselves are power-constrained machines. Using drones for IoT communications can be an expensive solution if they are not optimally positioned. The total cost of DBS deployment constitutes the flight time, the connection establishment time, disconnection time, and the cost of drones as a device. Therefore, it is of paramount importance to come up with an optimal drone placement to serve the maximum number of IoT devices. In the literature, the drone placement problem for different objectives is considered in a number of published works. In [11], the authors consider a 2D placement problem with an objective to maximize the network coverage while minimizing the transmit power. A heuristic is proposed to solve the optimization problem. The formulated 2D placement problem is further modified for efficient drones deployment in a 3D scenario. In [12], a backhaul-aware 3D drone placement is considered for a heterogeneous network. The placement algorithm maximizes the sum-rate and number of served users, considering the limiting factors, the peak data rate of wireless backhaul, and the bandwidth of DBS. In most of the research, the free-space path loss model has been considered for DBS deployment; however, in real scenarios, the IoT devices are scattered everywhere, that is, at indoor or outdoor locations. In [13], the wireless coverage for indoor users inside the high-rise buildings is considered. The presented model assumes a single drone. A hybrid path-loss model is proposed, which takes into account the path losses due to free-space, building penetration, and indoor losses. Particle swarm optimization (PSO) is generally used to minimize the total transmit power required to cover the indoor users. In [14], a solution was proposed to maximize the revenue of the cellular network using 3D placement of DBSs. The problem was formulated as a mixed-integer non-linear problem (MINLP) and solved using a heuristic. In [15], 3D deployment of DBS was considered to study energy-efficient communication in mobile ad hoc networks. The work proposes an optimal DBS placement that minimizes the  UAV-recall-frequency (UAV-RF), while taking into account the on-board circuit power. The authors also studied the effect of scattering on the optimal hovering altitude of DBS. Furthermore, the work concludes that minimum UAV-RF is achieved when the transmit power is equal to the power consumed by the on-board circuit. Network life can be prolonged by limiting this on-board circuit power. In [16], a mathematical model is proposed for the optimal number of drones, along with their efficient placement in wireless cellular networks. The problem is solved using a PSO algorithm. In [17], a heuristic solution was proposed that minimizes the number of DBSs to provide wireless coverage to a group of distributed TBSs. In [18], the placement of DBSs was studied to maximize the coverage for users with different QoS requirements. In [19], a method was proposed for joint radio resource allocation, 3D placement, and user association of DBSs for IoT networks. As discussed earlier, drones can be used for the assistance of already deployed networks. In [20], the resource allocation problem for drone-assisted networks was investigated, where the drones were utilized to harvest energy to the D2D pairs. Similarly, in [1], the drones were adopted to assist the TBSs for densification. In [21], the authors proposed a methodology for UAV trajectory design and radio resource assignment for a vehicle-to-anything scenario. The work proposes a joint scheduling strategy where a vehicle can be served by both the terrestrial BS (TBS) or DBS, and the one that provides maximum throughput is selected. The TBSs are selected on the basis of round robin scheduling, whereas the DBS are selected using both round robin and proportionality fair scheduling. A heuristic solution is proposed for UAV trajectory planning. DBSs can be used in a distributed and multi-layered architecture, such as the micro-cell/picocell, macrocell/microcell, or just macrocell [22]. The DBSs can also function in cooperation, using swarm formation. But as the DBSs have limited computational, storage, and communication capacities, they are unable to perform computationally intensive tasks. In order to extend the capacity of DBSs swarms, an DBSs-edge-cloud computational model [23] was adopted in order to support resource-intensive application, such as big data, artificial intelligence, and computer vision. An on-demand UAV placement was proposed in [24], where the users were arbitrarily distributed. The problem was modelled as a Knapsack-like problem, where a density-aware placement algorithm was proposed to maximize the user coverage subject to the constraint of the minimum required data rates per user.

In [25], a joint optimization problem was solved for the placement and power allocation (PA) to improve the performance of the NOMA-UAV network. The objective was to maximize the sum rate of users. Along with IoT, the body sensor network (BSN) is also among one of the major applications of 5G networks. Both in IoT and BSN, the nodes are very quickly drained due to a number of performed operations, including registration, removal, and routing. Therefore, an energy-efficient solution is required to maintain a high transmission capacity. In [26], an efficient approach for device discovery in 5G-based IoT and BSNs using multiple UAVs was proposed, where significant achieved gains in energy consumption were claimed. An integrative IoT platform is presented in [27], where the drones are utilized to serve different IoT devices. [2] studies the role of drones as relays, both for amplify-and-forward (AF) and decode-and-forward (DF) protocols in the cooperative communication.

To the best of our knowledge, in most existing studies, one aspect of resource allocation is addressed, in that there are works which focus on DBS assignment alone, or works in which user association is optimized given a fixed deployment of DBSs. In contrast, this work proposes a mathematical model that jointly optimizes the DBS placement and resource allocation problem.

### 1.2. Contribution

The main contributions to this work are as follows.

- Firstly, a mathematical model is presented that jointly optimizes the resources used by IoT devices. The objective of the optimization problem is to minimize the ratio of the number of DBSs to the connected IoT devices. The proposed model is developed by adopting a realistic air-to-ground (A2G) path loss model;
- The formulation of the proposed model considers the practical network constraints, which include: (a) the DBS deployment budget, (b) the network’s QoS requirements, and (c) the DBS battery-life prolongevity;
- A low-complexity heuristic is proposed to solve the optimization problem;
- The simulation results are carried out for a number of network scenarios and validate the accuracy of the proposed solution. The results’ comparisons show that the proposed algorithm’s performance is close to that of the optimal solution.

## 2. System Model and Problem Formulation

**Figure 1:**
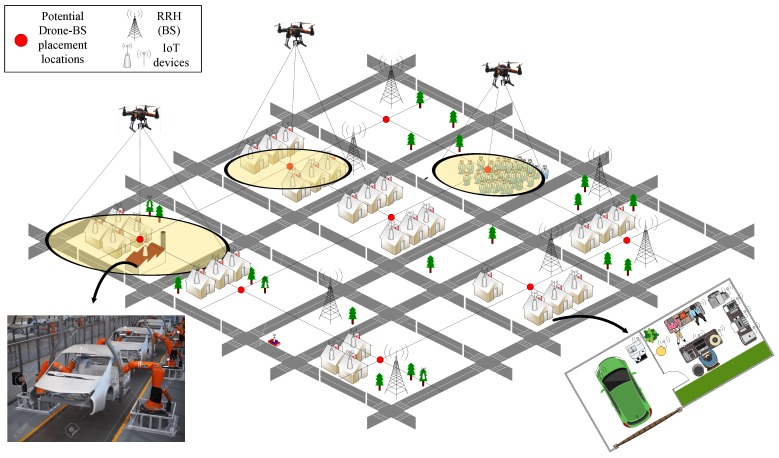
Proposed system model of an Internet of Things (IoT) network spread. It consists of *N* heterogeneous IoT (mobile and stationary) users spread over a metropolitan area with *M* potential locations for DBS placement.

Figure 1 illustrates an example of a massive IoT network spread over a metropolitan city. In practical situations, hundreds of heterogenous IoT devices are deployed over large geographical areas. These devices perform tasks in a wide variety of applications, including smart homes, remote asset control in industries, connected vehicles, wearable gadgets, smart shops, smart airports, tele-medicine, environmental monitoring, and disaster management. Such massive IoT deployment results in enormous growth of internet traffic, and requires existing cellular networks to undergo major architectural changes.

In a general IoT network, the IoT user equipments (UEs) are able to communicate with each other through wireless resources provided by TBSs, equipped with remote radio heads (RRHs). The RRHs are then connected to each other through high-speed communication links. Due to their mobility, it may be that a massive number of IoT devices are active in a given geographical area for a limited time. In such a situation, the DBSs come handy and provide a cost-effective solution to complement the TBSs and improve the connectivity and energy efficiency of IoT networks. DBSs can also help to restore communications in areas where terrestrial infrastructure has been collapsed after a disastrous situation.

Consider an IoT network which serves a total of *N* IoT devices  (In the following text, the word IoT devices is used interchangeably with IoT users or simply users) in a given geographical area. The area is divided into *M* potential candidate locations where DBS can be deployed. Due to their mobility, DBSs are able to move from one location to another in order to fulfil the connectivity requirements. Let’s say the triplet $\left(\bar{x_{i}},\bar{y_{i}},h_{i}\right)$ denotes the location of DBS *i* in a 3D system, where $\bar{x_{i}},\bar{y_{i}}$ are its position coordinates in a *X*-*Y* coordinate system, and $h_{i}$ is its height. This work assumes a constant height *h* for all DBSs deployed in the network. A DBS can potentially occupy any discrete location between (0,0,h) and $\left({\bar{x}}_{max},{\bar{y}}_{max},h\right)$.

The DBS placement algorithm is aimed at determining the optimal number and position of DBSs which maximizes the connectivity for a given set of IoT users. An air-to-ground (A2G) path loss model is assumed in this work, and discussed as follows.

### 2.1. Air-to-Ground (A2G) Path Loss Model

As shown in Figure 1, the exact locations of the IoT devices and the approximate locations of the obstacles, such as high-rise buildings, are known, along with the environment (e.g., urban, rural, suburban). However, due to a lack of complete information, the probabilistic approach is adopted. The probability of line-of-sight (LoS) and non-line-of-sight (NLOS) between DBSs placed at location *m* and the IoT device at *n* are given as [28]:

(1) $$\begin{array}{cc} P_{LoS}^{nm} & =\frac{1}{1+a\exp(-b\left[\theta_{mn}-a\right])},\;\forall m,n \end{array}$$

(2) $$\begin{array}{cc} P_{NLoS}^{mn} & =1-P_{LoS}^{mn},\;\forall m,n \end{array}$$

where *a* and *b* are the constants, and their values depend on the carrier frequency $f_{c}$ and type of environment, such as rural, urban, or dense urban [28]. $\theta_{mn}$ is the elevation angle between the DBS *m* and the IoT device *n*, and is equal to $\tan^{-1}\frac{h}{r_{mn}}$, where *h* is the altitude of DBS and $r_{mn}$ is the horizontal distance between DBS *m*, and the IoT device *n* is given as:

(3) $$r_{mn}=\sqrt{{\left({\bar{x}}_{n}-{\bar{x}}_{m}\right)}^{2}+{\left({\bar{y}}_{n}-{\bar{y}}_{m}\right)}^{2}+h^{2}},\;\forall m,n$$

The expressions for path loss for LOS and NLOS communication are given as $L_{LoS,mn}=Y+\sigma_{LoS}$ and $L_{NLoS,mn}=Y+\sigma_{NLoS}$, respectively. Here, $Y=10\alpha \left(\frac{4\pi f_{c}r_{mn}}{c}\right)$ [28], where *c* is the speed of light, α is the path-loss exponent, and $\sigma_{LoS}$ and $\sigma_{NLoS}$ are average additional losses which occur in addition to the free-space propagation loss. The values of $\sigma_{LoS}$ and $\sigma_{NLoS}$ depend on the environment. Finally, the average path loss can be represented as a function of DBS height and coverage circle, as:

(4) $$L\left(x_{m},y_{m},h_{m}\right)=P_{LoS}^{mn}L_{LoS}^{mn}+P_{NLoS}^{mn}L_{NLoS}^{mn},\;\forall m,n$$

and the average channel gain can be computed as:

(5) $$g_{mn}={L(x_{m},y_{m},h_{m})}^{-1},\;\forall m,n$$

Using the channel gain computed above, the signal-to-noise ratio (SINR) for a downlink channel between a DBS *m* and IoT user *n* can be computed as:

(6) $$SINR_{mn}=\frac{P_{m}g_{mn}}{\sum_{u\in I_{int}}P_{u}g_{un}+\sigma^{2}}$$

where $P_{m}$ is the transmit power of DBS *m*, $I_{int}$ is the set of interfering DBSs, and σ is the background noise.

### 2.2. Variable Definition

The main notations and variables used in this work are listed in Table 1. This work considers an IoT network composed of *N* IoT devices and *M* candidate locations for DBS placement. Let’s say x is a DBS placement vector of size *M*, given as:

(7) $$x=\left(x_{1},x_{2},\cdots ,x_{M}\right)$$

where an entry $x_{i}$ in x is a binary “1” when a DBS is placed at location *i*, and “0” otherwise. Similarly, let’s say Y is the user-DBS connectivity matrix of size N×M. An entry $y_{nm}$ of Y is a binary “1” when a user *n* is connected to a DBS placed at potential location *m*, and “0” otherwise.

### 2.3. System Constraints

The DBS deployment problem modelled in this work considers the following practical constraints.

#### 2.3.1. Minimum Users Connectivity Constraint

To achieve the QoS requirements of the IoT network, a certain minimum percentage β of total IoT devices must be served. Mathematically,

(8) $$\sum_{n=1}^{N}\sum_{m=1}^{M}y_{mn}\geq \beta N$$

where β∈[0,1].

#### 2.3.2. User Connectivity with Deployed DBS Constraint

According to this constraint, an IoT user must be connected to a potential location where DBS has actually been deployed. Mathematically,

(9) $$y_{mn}\leq x_{m},\;\forall n\in \left\{1,\cdots ,N\right\},m\in \left\{1,\cdots ,M\right\},$$

Equation (9) means that if a DBS is placed at a certain location, *m* (i.e., $x_{m}$ = ‘1’ in x), then a user may or may not be connected to it (i.e., entries in the *m*-th row of Y can be “0” or “1”). However, if a DBS is not placed at location *m* (i.e., $x_{m}$ = ‘0’), then no user can be connected to this location (i.e., all entries in row *m* of Y must be equal to “0”).

#### 2.3.3. Single DBS Connectivity for a User Constraint

A user/device can be connected to one DBS at most. Mathematically, this constraint can be formulated as:

(10) $$\sum_{m=1}^{M}y_{mn}\leq 1,\;\forall n$$

since rows of Y correspond to the DBSs and its columns correspond to users, $\sum_{m=1}^{M}y_{mn}$ indicates the number of DBSs to which the user *n* is simultaneously connected. According to (10), this sum must be less than or equal to “1” for each IoT user in the network.

#### 2.3.4. Maximum and Minimum Capacity of DBS Constraint

Each DBS in the network is a resource-limited device, and is capable of serving a certain maximum number $\gamma^{max}$ of users. Mathematically:

(11) $$\sum_{n=1}^{N}y_{mn}\leq \gamma^{max}x_{m},\;\forall m\in \left\{1,\cdots ,M\right\}$$

According to Equation (11), if a DBS is assigned at location *m* (i.e., $x_{m}$ = ‘1’), then the total number of users connected to it (i.e., $\sum_{n=1}^{N}y_{mn}$) must be upper-bounded by $\gamma^{max}$. Similarly, a DBS must be connected to a certain minimum number $\gamma^{min}$ of users to justify its deployment cost. Mathematically:

(12) $$\sum_{n=1}^{N}y_{mn}\geq \gamma^{min}x_{m},\;\forall m\in \left\{1,\cdots ,M\right\}$$

#### 2.3.5. DBS Deployment Budget Constraint

The DBS deployment incurs a cost, which includes the costs of flight time and connection establishment. The total deployment cost of all DBSs assigned must not exceed a certain available budget. Mathematically, this constraint can be modeled as:

(13) $$\sum_{m=1}^{M}c_{m}x_{m}\leq C^{max}$$

where $C^{max}$ is the maximum available budget for DBS deployment, and $c_{m}$ is the deployment cost of a single DBS placed at location *m*.

#### 2.3.6. Maximum User-DBS Distance Constraint

A user can only be connected to a DBS if its distance to the DBS location lies within a certain maximum value $d^{max}$. Mathematically,

(14) $$y_{mn}\cdot d_{mn}\leq d^{max}$$

where $d_{mn}$ denotes the distance between an IoT device *n* and the DBS placed at location *m*.

### 2.4. An Optimization Model for Drone-BS Deployment for Maximal Coverage

Given the network scenario and practical constraints mentioned above, this work considers the following mathematical model for DBS placement.

(15) $$\begin{array}{c} \begin{array}{cc} \min_{\begin{array}{c} x_{m}\in \left\{0,1\right\},\\ y_{mn}\in \left\{0,1\right\},\\ \forall n,m \end{array}} & \frac{\overset{\mathrm{No}.\;\mathrm{of}\;\mathrm{DBSs}}{\overbrace{\sum_{m=1}^{M}x_{m}}}}{\underset{\mathrm{No}.\;\mathrm{of}\;\mathrm{connected}\;\mathrm{IoT}\;\mathrm{devices}}{\underbrace{\sum_{n=1}^{N}\sum_{m=1}^{M}y_{mn}}}}\\ \mathrm{subject}\;\mathrm{to}: & \\ & \mathrm{Constraints}\;(8)–(14) \end{array} \end{array}$$

The optimization problem of (15) is an integer fractional optimization model. The numerator of the objective function denotes the total number of DBSs deployed, whereas the denominator denotes the total number of IoT users served by the network. Therefore, the optimization goal is to obtain maximum network connectivity with the minimum number of DBSs, while satisfying the practical network constraints.

### 2.5. Linearization of Optimization Problem

This work linearizes the fractional optimization problem of (15) using the method proposed in [29]. Let us consider two variables $t={\left\{\sum_{n=1}^{N}\sum_{m=1}^{M}y_{mn}\right\}}^{-1}$ and $\alpha =\max\left[1,{\min(\sum_{n=1}^{N}\sum_{m=1}^{M}y_{mn})}^{-1}\right]$, where $y_{mn}\in \left\{0,1\right\}$ and $\sum_{n=1}^{N}\sum_{m=1}^{M}y_{mn}>0$. Therefore, α=max[1,1]=1. Now the optimization problem (15) can be rewritten as:

(16) $$\begin{array}{c} \begin{array}{cc} \min_{\begin{array}{c} x_{m}\in \left\{0,1\right\},\\ y_{mn}\in \left\{0,1\right\},\\ 0\leq t\leq 1\\ \forall n,m \end{array}} & \;\;\alpha \sum_{m=1}^{M}x_{m}t\\ \mathrm{subject}\;\mathrm{to}: & \\ & \mathrm{Constraints}\;(8)–(14)\\ & \alpha \sum_{n=1}^{N}\sum_{m=1}^{M}y_{mn}t=1 \end{array} \end{array}$$

The objective function in (16) can be expanded as,

(17) $$\min\alpha [x_{1}t+x_{2}t+\ldots +x_{M}t]$$

and constraint $\alpha \sum_{n=1}^{N}\sum_{m=1}^{M}y_{mn}t=1$ in (16) can be expanded as,

(18) $$\begin{array}{cc} \min & \;\;\alpha [y_{11}t+y_{12}t+\ldots +y_{1N}t+y_{21}t+\ldots +y_{2N}t+\ldots +y_{M1}t+\ldots y_{MN}t]=1 \end{array}$$

Let’s say that $z_{1}=x_{1}t$, $z_{2}=x_{2}t$, …, $z_{M}=x_{M}t$ and $w_{11}=y_{11}t$, $w_{12}=y_{12}t$, …, $w_{MN}=y_{MN}t$. As shown in [29], the multiplication of a binary variable *a* with a continuous variable *b*, that is, c=ab, can be represented as linear inequalities, such as:

(19) $$c=ab\Rightarrow \left\{\begin{array}{c} \left(i\right)\;b-c\leq 1-a\\ \left(\mathrm{ii}\right)\;c\leq b\\ \left(\mathrm{iii}\right)\;c\leq a\\ \left(\mathrm{iv}\right)\;c\geq 0 \end{array}\right.,$$

In (17) and (18), there are *M* and M+N multiplications of the continuous variable *t* with binary variables $x_{m}$ and $y_{mn}$, respectively. By using (19), these multiplications can be converted into linear inequalities. Therefore, the optimization problem (16) can be restated as:

(20) $$\begin{array}{c} \begin{array}{cc} \min_{\begin{array}{c} x_{m},y_{mn}\in \left\{0,1\right\},\\ z_{m},w_{mn}\geq 0,\\ 0\leq t\leq 1\\ \forall n,m \end{array}} & \;\;\alpha \sum_{m=1}^{M}z_{m}\\ \mathrm{subject}\;\mathrm{to}: & \\ & \mathrm{Constraints}\;(8)–(14)\\ & \alpha \sum_{n=1}^{N}\sum_{m=1}^{M}w_{mn}=1\\ & t-z_{m}\leq 1-x_{m},\;\;\forall m\\ & z_{m}\leq t,\;\;\forall m\\ & z_{m}\leq x_{m},\;\;\forall m\\ & t-w_{mn}\leq 1-y_{mn},\;\;\forall m,\;n\\ & w_{mn}\leq t,\;\;\forall m,\;n\\ & w_{mn}\leq y_{mn},\;\;\forall m,\;n \end{array} \end{array}$$

## 3. Proposed Solution

The linear programming optimization problem of (20) is solvable optimally by using a Branch and Bound (B&B) algorithm. However, the worst case complexity is exponential to the product of the sizes of the binary variables x and Y. This work proposes a low-complexity greedy heuristic for joint DBS placement and the user association problem. Algorithm 1 shows the main computation steps of the proposed solution, whose main symbols and notations are listed in Table 1. The algorithm receives as inputs, the values of $N,M,\gamma^{max},\gamma^{min}$, and $d^{max}$. For simulation purposes, the network area is represented as a grid with discrete potential DBS locations, and where the users are evenly distributed. A matrix d of dimensions N×M is obtained, whose entry $d_{nm}$ denotes the distance of an IoT user *n* from DBS location *m*. During the initialization phase, the important variables are initialized, which include the DBS placement vector x, user-DBS association matrix Y, a vector c of size *M* which denotes the number of users served by each DBS, and a vector t of size *N* which denotes the number of DBSs connected to each user. A binary matrix W of dimensions N×M is also computed, which shows all possible connections between users and DBSs. An entry $w_{n,m}$ of W is equal to “1” only if the distance between the user *n* and location *m* is less than the maximum distance $D^{max}$, and is “0” otherwise. The execution of the proposed algorithm is divided into five phases, which are discussed below.

**Algorithm 1** Heuristic for efficient drone placement.
1:
**Inputs:** $N,M,\gamma^{max},\gamma^{min},\beta ,R,d^{max}$
2:
**Initialization:** ∀n∈{1,⋯,N},m∈{1,⋯,M}
3:$Y_{nm}\leftarrow 0$, $x_{m}\leftarrow 0$, $c_{m}\leftarrow 0$, $t_{n}\leftarrow 0$4:
$W_{nm}\leftarrow \left\{\begin{array}{cc} 1, & \mathrm{if}\;R_{nm}<d^{max}\\ 0, & \mathrm{otherwise} \end{array}\right.$
5:
**Step I: Allocation to users with single**

**connection option**
6:
$t_{n}\leftarrow \sum_{n=1}^{N}W_{n,m^{\prime }},\;\forall m^{\prime }\in \left\{1,\cdots ,M\right\}$
7:
**for** i∈{1,⋯,N} **do**
8: **if** $t_{i}==1$ **then**
9:  $k\leftarrow find(W_{i,:}==1)$
10:  $Y_{i,k}\leftarrow 1,\;x_{k}\leftarrow 1,\;c_{k}\leftarrow c_{k}+1$
11: **end if**
12:
**end for**
13:
**Step II: Allocation of users with connections**

**options to multiple drones**
14:
**for** i∈{1,⋯,N} **do**
15: **if** $t_{i}>1$ **then**
16:  flag←0
17:  $k\leftarrow find\left(W_{i,:}==1\right),\;j\leftarrow find\left(x==1\right)$
18:  [I,λ]←sort(c)
19:  **for** j=1:len(I) **do**
20:   $a_{j}\leftarrow \left\{\begin{array}{cc} 1, & \mathrm{if}\;I_{j}\in k\\ 0, & \mathrm{otherwise} \end{array}\right.$
21:  **end for**22:  **if** $\sum_{j=1}^{len(a)}a_{j}>0$ **then**
23:   $I^{\prime }\leftarrow find\left(a==1\right)$
24:   **for** $q\leftarrow 1:len(I^{\prime })$ **do**
25:    $s\leftarrow I_{q}^{\prime }$
26:    **if** $c_{I_{s}}<\gamma^{max}$ **then**
27:     $Y_{i,I_{s}}\leftarrow 1,\;x_{I_{s}}\leftarrow 1,\;c_{I_{s}}\leftarrow c_{I_{s}}+1$
28:     flag←1
29:     break30:    **end if**
31:   **end for**
32:   **if** flag==0 **then**
33:    k←k∖I
34:    **if** k==∅ **then**
35:     $W_{\left(i,:\right)}\leftarrow 0$
36:     flag←1
37:    **end if**
38:   **end if**
39:  **end if**
40:  **if** flag==0 **then**
41:   $Y_{i,k_{1}}\leftarrow 1,\;x_{k_{1}}\leftarrow 1,\;c_{k_{1}}\leftarrow c_{k_{1}}+1$


42:  **end if**
43: **end if**
44:
**end for**
45:
**Step III: Check for under utilized drones**
46:
$\nabla \leftarrow find(\left(\left(c<\gamma^{min}\right)\cdot x\right)==1)$
47:
$Y_{:,\nabla }\leftarrow 0,\;x_{\nabla }\leftarrow 0,\;c_{\nabla }\leftarrow 0$
48:
$p\leftarrow \sum_{i=1}^{N}Y_{i,:}$
49:
$u^{\prime }\leftarrow find\left(p==0\right)$
50:
b←round(β·N)
51:
$t\leftarrow \sum_{i=1}^{N}\sum_{j=1}^{M}Y_{i,j}$
52:
**for** $i\leftarrow 1:len(u^{\prime })$ **do**
53:
**if** t<b **then**
54: flag←0
55:  [I,λ]←sort(c)
56:  **for** j←1:M **do**
57:   **if** flag==0 **then**
58:    **if** $c_{\lambda_{j}}<\gamma^{max}\mathrm{and}W_{u_{i}^{\prime },\lambda_{j}}==1$ **then**
59:     $Y_{u_{i}^{\prime },\lambda_{j}}\leftarrow 1,\;x_{\lambda_{j}}\leftarrow 1,\;c_{\lambda_{j}}\leftarrow c_{\lambda_{j}}+1$
60:     flag←1
61:     $t\leftarrow \sum_{ii=1}^{N}\sum_{jj=1}^{M}Y_{ii,jj}$
62:    **end if**63:   **end if**
64:  **end for**
65: **end if**
66:
**end for**
67:
**Step IV: Connect all unconnected users**
68:
$t\leftarrow \sum_{i=1}^{N}\sum_{j=1}^{M}Y_{i,j}$
69:
$p\leftarrow \sum_{i=1}^{N}Y_{i,:}$
70:
$u^{\prime }\leftarrow find\left(p==0\right)$
71:
**for** $i\leftarrow 1:len(u^{\prime })$ **do**
72: **if** t<b **then**
73:  $r\leftarrow R_{\left(i,:\right)}$
74:  flag←0
75:  $k\leftarrow W_{u_{i}^{\prime },:}$
76:  **for** j←1:len(k) **do**77:   **if** flag==0 **then**
78:    $\left[l^{v},l^{i}\right]\leftarrow \min\left(r\right)$
79:    $r_{l^{i}}\leftarrow \inf$
80:    $\left[l^{v},l^{i}\right]\leftarrow \min\left(r\right)$
81:    **if** $W_{u_{i}^{\prime },l^{i}}>0$ **then**
82:     $Y_{u_{i}^{\prime },l^{i}}\leftarrow 1,\;x_{l^{i}}\leftarrow 1,\;c_{l^{i}}\leftarrow c_{l^{i}}+1$
83:    **end if**
84:   **end if**
85:  **end for**
86: **end if**
87:
**end for**
88:
**Step V: Remove all under-utilized drones**
89:
$d^{u}\leftarrow find\left(\left(c<\gamma^{min}\right)\cdot x==1\right)$
90:
$Y_{:,d^{u}}\leftarrow 0,\;x_{d^{u}}\leftarrow 0,\;c_{d^{u}}\leftarrow 0$
91:
$t\leftarrow \sum_{i=1}^{N}\sum_{j=1}^{M}Y_{i,j}$
92:
**Outputs:** Y,x



### 3.1. Phase I: Allocation of Users within the Coverage Area of a Single DBS

In the first phase, those users are assigned which are located within the coverage area of a single DBS location. Step 8 obtains the total number of DBS connections possible for each user n∈{1,⋯,N}. This is done by adding all entries at row *n* of W and storing the result at the corresponding row in vector t. In the next step, for the loop runs for *N* iterations, if the possible number of DBS connections of user *i* (i.e., $t_{i}$) is equal to “1” during each iteration, then the corresponding index of DBS is obtained from W (i.e., column index) and stored in *k*. The *k*-th entry in x is set to “1” to mark a DBS placement at potential location *k*. Similarly, the entry $y_{i,k}$ is set to “1” in Y to mark an association between user *i* and DBS location *k*. Finally, the count value of connected users for DBS *k* is updated in c.

### 3.2. Phase II: Allocation of Users within the Coverage Area of Multiple DBS Locations

During this phase, those users are assigned which are located in the coverage area of multiple DBS locations. In Step 17, for loop runs for *N* users (iterations), for each user *i*, the number of its potential DBS connections (i.e., $t_{i}$) is obtained. If this value is greater than “1”, then Step 20 obtains the indexes of all potential DBSs for user *i* and stores them in vector k. In addition, the indexes of those DBSs already deployed are obtained from x and stored in vector j. The function find(·) returns the indexes where the argument is true. In Step 21, the non-zero entries of c are sorted in descending order and stored in vector λ along with their indexes in vector I. In Steps 22–24, a binary vector a is obtained which has non-zero entries for the indexes of all DBSs which have already been deployed, and can also be assigned to user *i*. The indexes of these DBSs are obtained in Step 26. Among these potential DBSs for user *i*, the DBS connected to maximum number of users is selected, provided it satisfies the maximum capacity $\gamma^{max}$ criteria. Such user-to-DBS assignment is done in Steps 26–34, along with the update of variables x,Y, and c. A temporary flag variable is set whenever a user is assigned. If all the existing DBSs are fully up to their maximum capacity, then the flag is still 0 at the end of Step 34. Steps 35–42 exclude the indexes of deployed DBSs from the potential connections for user *i*. If the result is a null vector ϕ, it means that the user *i* cannot be connected to any DBS other than existing DBSs which are already full. Such a user is not assigned by the network as shown in Step 38, which places a “0” at all DBS indexes in W for user *i*. In the third case, a user may have potential connection to one or multiple locations where no DBS has already been deployed. In such a case, a new DBS is placed at the location with the smallest available index, and user *i* is associated to it. This is done in Steps 43–47 by updating the corresponding entries of x, Y, and c.

### 3.3. Phase III: Check for Under-Utilized Drones

In Step 50, the indexes are stored in **∇** for all DBSs that remain under-utilized after the association steps of phase I and II. These DBSs are removed in Step 51 by disassociating their connected users and setting the relevant entries of Y, x, and c to “0”. In Steps 52–53, the indexes of unconnected users are obtained and stored in vector $u^{\prime }$. Step 54 computes *b*, that is, the minimum number of users that must be serviced, whereas Step 55 computes *t*, that is, the number of users that have been serviced so far. If the number of served users is less than the minimum users to be served, it is executed until both these numbers become equal. During each iteration of the loop, that is, in Steps 56–70, one of the unconnected users is selected from the list $u^{\prime }$ and assigned to an existing DBS with the highest utilization, with the condition that the DBS is not overcrowded and belongs to the set of DBSs that have potential connections for the user. The user-DBS association is marked by updating the relevant entries of Y, x, and c.

### 3.4. Step IV: Association of All Unconnected Users until Now

In this phase, the users which remain unconnected in the previous phases are associated to the DBSs. Their indexes are obtained in $u^{\prime }$ in Step 75. The for loop of Steps 76–92 performs user-DBS association on the basis of distance. For each user $i\in u^{\prime }$, its distances from all the DBS locations are obtained from matrix R and stored in r in Step 78. In Step 79, the list of potential DBS connections for the users is obtained and stored in k. In Steps 82–86, among all potential DBSs for the user, the DBS with the minimum distance is selected. The user association to this DBS is marked in Step 87 by updating the relevant matrices.

### 3.5. Step V: Remove All Under-Utilized Drones

Finally, the under-utilized drones are removed by disassociating the users connected to them, and the total number of connected users is computed. The outputs of the algorithm are the user-DBS association matrix Y and the DBS placement vector x.

### 3.6. Complexity

The main advantage of the proposed method is its low computational complexity, where the complexity is calculated in terms of flops. The complexity of the initialization phase (Steps 2–4) is 5MN. The complexity of Steps 6–12 is 2MN+4N. The worst case complexity of Steps 14–44 is $M^{2}N+11MN+13N$, and Steps 46–66 have a worst case complexity equal to $2M^{2}+M^{2}N+9M$, where Steps 68–87 have a complexity 6MN+9N and Steps 89–91 have a complexity equal to 5M+MN flops. The worst case complexity of the proposed heuristic overall is $0(M^{2}N)$, whereas the complexity of the optimal algorithm is $2^{NM^{2}}$. This clearly demonstrates that the proposed solution is fairly applicable for practical, large-area IoT networks.

## 4. Results and Discussion

This section presents the simulation results of proposed solution. As discussed earlier, the network area is visualized as a grid with discrete points which represent the potential DBS locations, and the users are evenly distributed on the grid. The simulations are carried out for a variety of realistic network scenarios with grid sizes of 3×3, 4×4, and 5×5 DBS locations, with the total number of users *N* ranging from 50 up to 200 and β=0.2 and 0.8. The optimization goal is to minimize the utility function of (15), which is represented as a ratio between the number of deployed DBSs and the number of connected users. The performance of the proposed heuristic is compared with the optimal algorithm. As discussed earlier, β is the minimum fraction of users that must be serviced. This means that for a larger β, more drones need to apparently be deployed. Similarly, if the grid size is smaller, it would be difficult to service a larger β percentage of users. These facts have been proven through a number of simulations, and the results are discussed below.

Before going into the simulation analysis, let’s try to analyze the objective function. The objective function is the ratio of the total number of deployed drones to the total number of connected users. The objective is to minimize this ratio. From the basic mathematical theory, it can be easily observed that this ratio (objective function) could be minimized if:

- The numerator is decreased, keeping the denominator fixed;
- The denominator is increased, keeping the numerator fixed;
- The numerator is decreased, and at the same time, the denominator is increased.

In this objective function, the numerator represents the total number of deployed drones, whereas the denominator represents the total number of connected users. Therefore, from the analysis above, either a smaller number of drones have to be deployed, keeping a fixed number of connected users, or more users need to be connected with a smaller number of drones. The third option is to decrease the number of drones, and increase the number of connected users at the same time.

**Figure 2:**
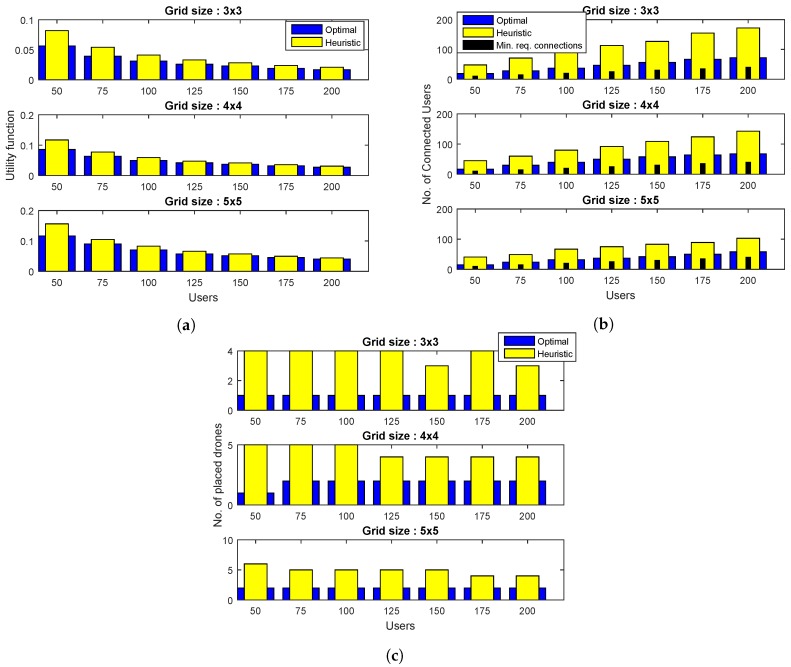
Comparison of proposed heuristic with the optimal algorithm for β=0.2 and different grid sizes, in terms of: (**a**) The total number of connected users, (**b**) total number of placed drones, and (**c**) the utility value, that is, the ratio of the total number of placed drones to the total number of connected users.

In Figure 2, the performance results are reported for different grid sizes and β=0.2. Figure 2a plots the value of objective function (15) for different grid sizes and the number of users. A number of trends can be observed from the plot. The value of objective function achieved by optimal and heuristic algorithms increases with grid size, keeping other parameters constant. This is due to the fact that greater grid size means a greater number of potential locations. This increases the probability of greater DBS deployment. Moreover, for a given grid size, the value of the utility function and the performance gap between the two algorithms decrease with an increase in the number of users. This trend is due to the fact that as more users are placed within the network of a given grid size, the degree of freedom of their association with DBSs increases, resulting in better overall network utilization. For all grid sizes and the number of users, the utility function value obtained by the proposed heuristic is fairly close to the optimal solution.

In Figure 2b, the performance results are presented in terms of the number of connected users. For all grid sizes and values of *N*, both algorithms satisfy the minimum QoS requirements represented as black bars in the plots. The plots further reveal that, for a given grid size and *N*, the proposed heuristic results in greater number of connected users as compared to the optimal one. Moreover, the user connectivity achieved by the proposed heuristic decreases with an increase in grid size, whereas this quantity remains almost the same for an optimal algorithm. This trend is due to the fact that as grid size increases for a given value of *N*, the users initially get connected to their nearest DBS location, resulting in some drones being under-utilized. These DBSs are removed in the later phases of the algorithm to satisfy constraint C4. As a result, some of the users are connected to the existing DBSs, while others remain unassigned. In Figure 2c, the performance results are plotted in terms of the number of DBSs assigned. The proposed heuristic places more DBSs, as compared to the optimal algorithm. However, it also serves a greater number of users. Overall, the combined result, that is, the ratio of quantities of Figure 2b,c of the proposed heuristic, is comparable to the optimal solution.

**Figure 3:**
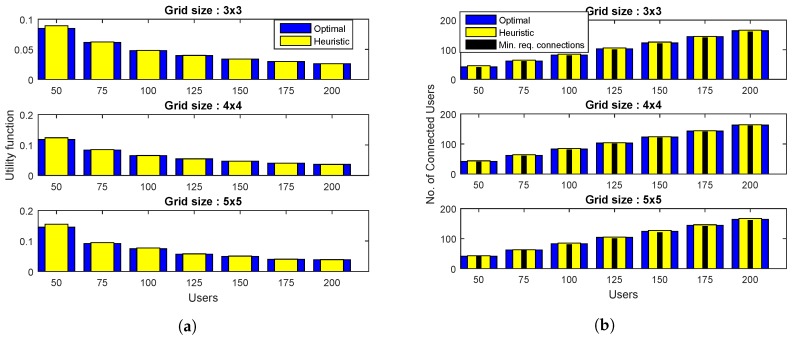

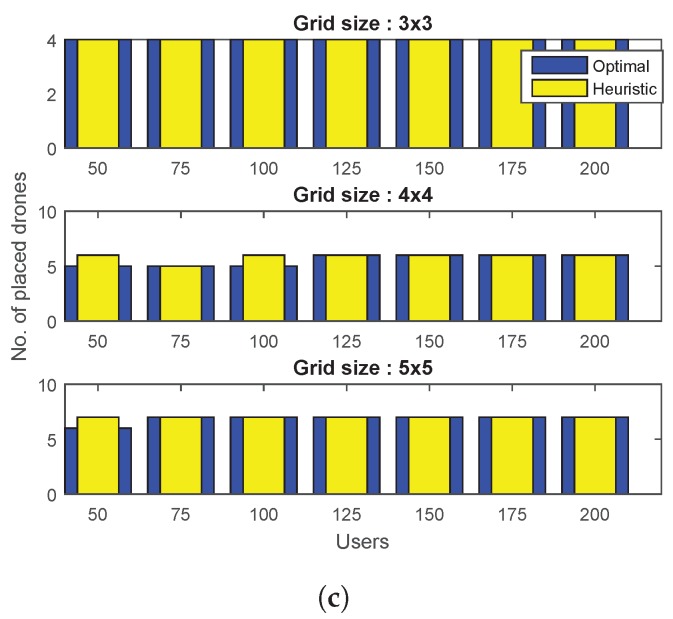
Comparison of proposed heuristic with the optimal algorithm for β=0.8 and different grid sizes in terms of: (**a**) The total number of connected users, (**b**) total number of placed drones, and (**c**) the utility value, that is, the ratio of the total number of placed drones to the total number of connected users.

Figure 3 demonstrates the simulation results for β=0.8 and various grid sizes. The value of utility functions for two algorithms are plotted in Figure 3a, which shows that both algorithms perform close to each other. A similar trend is exhibited by the plots of Figure 3b,c, where the proposed heuristic not only satisfies the minimum user connectivity requirements for all cases, but also achieves a number of connected users and DBSs that is fairly close to the optimal algorithm.

**Figure 4:**
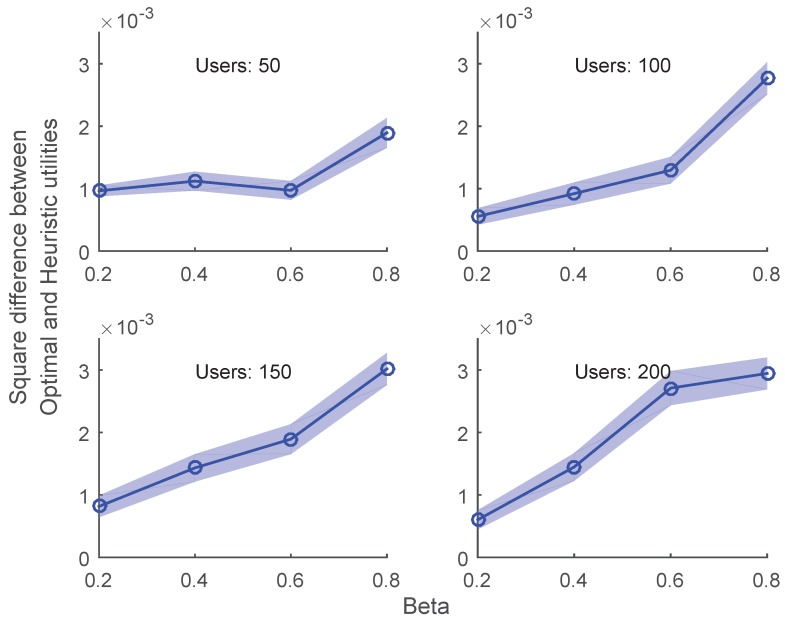
Confidence intervals for grid size =3×3.

Finally, in Figure 4, the confidence interval (CI) is plotted as a function of β for various values of *N*. CI is defined as the squared difference between the utility function value of the optimal and proposed heuristic. A very small value of CI is achieved for all cases of simulation, which validate the numerical accuracy of proposed scheme.

## 5. Conclusions

In this paper, DBSs have been investigated as a promising solution to fulfil the aggressive connectivity and QoS requirements of large-scale IoT networks. However, DBSs being power-constrained devices themselves, a careful scheme is required for their deployment and the user association to them. This aspect was addressed in this work, by first providing a realistic mathematical model for the joint optimization problem of DBS assignment and user association in a massive, large-scale IoT network. The mathematical model was developed by keeping in mind an air-to-ground path-loss model and practical network constraints. The objective function minimizes the ratio between the number of DBSs utilized to the number of users connected. Such an integer fractional problem suffers from prohibitive complexity when solved optimally for practical-sized networks. A linearization of the problem was proposed, along with a low-complexity heuristic algorithm for its solution in linear time. A comparison of the results of the proposed solution with the optimal one confirmed the accuracy and validity of our approach.

## Figures and Tables

**Table 1:** List of notations and variables of this work.

Notation	Explanation
*N*	Total no. of IoT users
*M*	Total no. of potential locations for DBS placement
x	Binary indicator vector of size *M* for DBS placement
Y	Binary matrix of size N×M, which indicates achieved associations between users and DBSs
β	Constant which indicates the minimum percentage of users that must be serviced by the network
$\gamma^{min}\left(\gamma^{max}\right)$	Minimum (Maximum) number of users that each DBS must (could) serve
$c_{m}$	Cost of DBS deployment at location *m*
$C^{max}$	Maximum deployment cost of all DBSs
$d_{nm}$	Distance between user *n* and DBS location *m*
$d^{max}$	Maximum coverage distance of a single DBS
W	A matrix of dimensions N×M which indicates potential DBS associations for each user
R	A matrix of dimensions N×M which contains the distance between IoT users and DBS locations
t	A column vector of size *N* indicating the possible number of DBS connections for each user
c	A row vector of size *M* which indicates the total number of connected users for each DBS
find(x)	Function which returns the indexes where its argument *x* is true
sort(c)	Sorts the vector *c* in descending order and returns values and indexes
**∇**	Vector of size *M* which stores the indexes of under-utilized DBSs
$u^{\prime }$	Vector which stores the indexes of unconnected users
len(x)	Returns the size of vector x
min(r)	Returns the minimum value and its indexes from the vector r
:	Operator indicating complete row or column in a matrix
